# Glycogen—Endoplasmic Reticulum Connection in the Liver

**DOI:** 10.3390/ijms24021074

**Published:** 2023-01-05

**Authors:** József Mandl

**Affiliations:** Department of Molecular Biology, Faculty of General Medicine, Semmelweis University, P.O. Box 2, 1428 Budapest, Hungary; jozsef.mandl@med.semmelweis-univ.hu

**Keywords:** glycogen, liver, endoplasmic reticulum, biotransformation, antioxidant defense, drug induced liver injury, glucuronidation

## Abstract

Glycogen, the branched polymer of glucose is found mainly in the liver and muscle in mammals. Along with several other proteins, glycogen forms separate cellular organelles, and particles in cells. Glycogen particles in the liver have a special metabolic and also regulatory connection to the intracellular endomembrane system, particularly the endoplasmic reticulum. This connection is part of the organelle homeostasis in hepatocytes and forms a “glycogenoreticular system”. The actual size of hepatic glycogen stores and the rate of glycogenolysis determines several essential liver-specific metabolic processes, such as glucose secretion for the maintenance of blood glucose levels or the glucuronidation of certain vital endo-, and xenobiotics, and are also related to liver antioxidant defense. In starvation, and in certain physiological and pathological states, where glycogen stores are depleted, functions of the glycogenoreticular system are altered. The starvation-induced depletion of hepatic glycogen content changes the biotransformation of various endo- and xenobiotics. This can be observed especially in acute DILI (drug-induced liver injury) due to paracetamol overdose, which is the most common cause of acute liver failure in the West.

## 1. Introduction

Since glycogen consists only of glucose molecules, it was long thought of as the most boring macromolecule in close competition with DNA. In those times DNA was considered as uniform, uninteresting, monotonous structural material, consisting of four types of building units, while glycogen was made up of only one type. However, by the middle of the twentieth century, DNA turned out to be the most exciting member of the world of the biologically important molecules, even compared to proteins. The development of biochemistry also fundamentally overwrote the ideas about glycogen being the most boring member of the macromolecule family.

Looking at this world from the point of view of “molecular logic”, glycogen metabolism is characterized by a highly advanced packaging technology and “warehouse logistics”. The discovery of glycogen storage diseases shed light on the effectiveness of the packaging technique of glucose molecules and on the cellular and physiological significance of the organization of rapid loading and unloading from storage. The molecular task of loading and unloading from storage requires the functioning and regulation of enzymes. Our appreciation of glycogen biochemistry has also changed from a pure carbohydrate biochemistry into one of the most exciting examples of enzyme regulation and cellular signaling. In the age of cellular organelle biochemistry, the glycogen molecule became a glycogen particle, and the role of organelle interactions in cellular molecular biology came to the fore. It is hard to understand the metabolic functions of glycogen without understanding the logic of cell–organelle relationships.

The organelle-organelle interrelationship is a fundamental part of cellular homeostasis. In this homeostasis, the endoplasmic reticulum (ER), the endomembrane system, plays a key role, and operates in dynamic, constantly changing relationships with each type of cellular organelle. The glycogen granules are one of these cell organelles, that are sometimes attached to or detached from the ER similar to other types of organelles. Non-covalent connections between macromolecules, membranes and organelles can be extremely decisive in the regulation of a series of processes at the molecular level (e.g., blood coagulation). Accordingly, glycogen degradation provides substrates for processes that take place in the luminal compartment of the ER, or sometimes in intralysosomal processes [1]. Organelle-organelle relationships, in which one of the participants is the glycogen particle also determine the metabolic fate of the glucose molecules stored in glycogen. The different organelles represent different metabolic compartments, so the glucose molecule leaving the glycogen store can be connected to different metabolic pathways.

Liver carbohydrate metabolism has several special functions also at the organism level, including its role in the maintenance of the blood glucose level. This article mainly focuses on two additional important liver metabolic functions in which glycogen metabolism plays a decisive role and in which the role of the glycogen particle–ER connection is not clearly understood: drug metabolism and antioxidant defense. The glycogen particle–ER connection is a basic attribute of glycogen metabolism, one that is essential for its role in drug metabolism and antioxidant protection.

## 2. Glycogen Molecule as Glucose Store and the Glycogen Particle as a Cellular Organelle in Hepatocytes

Glycogen forms separate cellular organelles. Besides the polysaccharide macromolecule, a range of different glycogen metabolism enzymes and other proteins are involved in glycogen particles [2,3,4,5,6].

Glycogen degradation provides fuel for different processes depending on cell types: contraction in the muscle, learning capacity in the brain, etc. [6]. Moreover, glycogenolysis provides also cofactors and precursors for certain processes in biotransformation and antioxidant defense in the liver [7]. However, glycogen is also found in various other cells and tissues such as erythrocytes, adipose tissue, heart, etc., where its functions are less characterized [8].

**Figure 1:**
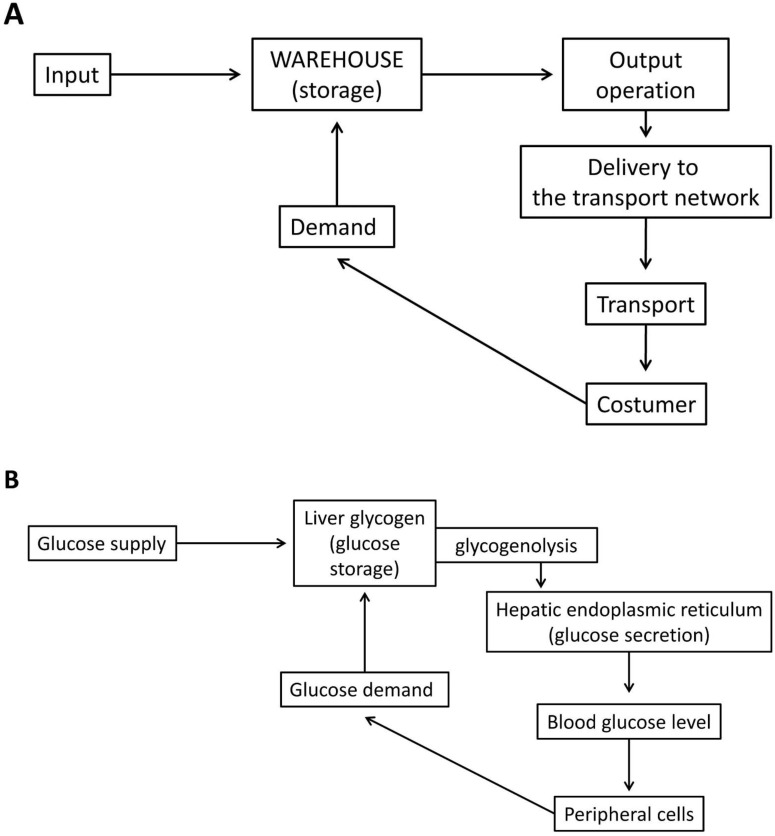
In the general logical scheme of warehousing, the product stored in the warehouse travels from the central warehouse to the consumer via a transport network. An important step in the sequence of events is the delivery of the product from the warehouse to the transport network (**A**). A huge amount of glucose molecules stored in form of glycogen can reach the peripheral cells via the bloodstream. The “output operation” is realized mainly through glycogenolysis. The necessary enzyme-machinery for glycogen degradation is part of the glycogen particle. However, the final step of the output operation occurs in another cell organelle, the endoplasmic reticulum (ER). This operation requires another enzyme reaction catalyzed by the ER membrane bound glucose-6 phosphatase enzyme—transporter system, thereafter glucose is secreted into circulation. ER is needed for establishing this connection between the storage molecule and the bloodstream (**B**).

Liver cells are able to secrete glucose, which can be consumed at various sites in the body. Therefore, the storage function of glycogen (the molecular “packaging” technology of the glucose units in glycogen: the structural biochemistry of the α-1,4-glycosidic and α-1,6-glycosidic bonds, or the biochemistry of fast glucose “warehouse loading and unloading”) is fundamentally different in the liver, because products from the central warehouse must reach remote consumers. Whenever necessary, the ER of the hepatocytes can raise the blood sugar level, at the expense of glucose released from the glycogen stores (Figure 1).

There exist a unique relationship between glycogen particles and the endomembrane system, particularly the ER membrane in the liver [1,7]. This relationship determines the kind of utilization of glucose stored in glycogen molecules. Several glycogen-dependent biosynthetic processes are accompanied by the participation of various hepatic ER membrane-bound enzymes and transporters. The ER is a separate metabolic compartment surrounded by special membranes, which is also part of the endomembrane vesicular transport system [9]. The ER is connected to every cellular organelle and is an ideal nutrient sensor. The ER is also able to respond to different metabolic changes and demands, among others to hypo- or hyperglycemia, and an increase in the exposition of foreign compounds. Thus, ER plays a prominent role in cellular adaptation to both the external and internal environment [10,11]. Glycogen particles and the ER can be combined also with phagophores forming autophagosomes. This way glycogen is degraded by acid alpha-glucosidase and not during glycogenolysis [1].

The ER has primarily biosynthetic functions in the cell [10,11]. However, other organelles are also needed for ER-related biosynthetic processes, e.g., ribosomes. It is well known that in the most energy-consuming anabolic process, protein synthesis, ribosomes are coupled to ER membranes. Smooth and rough ER are distinguished based on this organelle-organelle connection. As glycogen particles can be also attached to ER membranes, the various glycogenolysis-dependent hepatic biosynthetic processes are catalyzed by intraluminally oriented ER membrane enzymes, and also mediated by ER transporters [7]. The liver, being a special secretory organ exports various molecules of different molecular weights (e.g., lipoproteins, glucose, conjugated drug metabolism products, glutathione, ascorbate, and bile acids) formed in the luminal compartment of the ER and secreted by hepatocytes. Glucose, originating from glycogen particles affects these secretory, biosynthetic ER processes and becomes part of the product [10,11].

## 3. Moieties and Functional Roles of the ER-Glycogen Particle Relationship in the Liver

The glycogen content of the liver is a requisite for many liver functions, as it provides precursors or cofactors for different biosynthetic processes. Metabolism can be divided into glucogenic and ketogenic parts; consequently, there are glucogenic and ketogenic reserves. Glycogen, as a glucogenic reserve serves as a glucose or glucose-derived donor in several biosynthetic processes. The liver is able to secrete glucose into the blood to maintain the blood glucose level, which is a fundamental nutrient source for every cell of the body. Therefore, the preservation of liver glucogenic reserves is a priority at the level of the organism, of the organ and of the hepatocyte. In addition, the liver is also the organ in which detoxification occurs. Biotransformation enzymes that detoxify the body from xenogenic substances and also from many molecules produced in the body are mainly expressed in the liver. Drug metabolism is essentially a biosynthetic process because the substances that we can remove from the body in different forms must first be synthesized. Products of biotransformation reactions are formed in the course of drug metabolism. The process of transformation, which takes place during drug metabolism is a prerequisite for drugs to be secreted from the hepatocytes into the blood or bile and eliminated in various ways from the organism. Glucuronidation is a high-capacity form of biotransformation, which enable the excretion of potentially toxic endo- and xenobiotics, such as bilirubin and also various drugs, etc. UDP glucuronic acid, a cofactor for glucuronide formation is formed at the expense of glycogen stores [12]. Moreover, there is a complex coordinated regulatory relationship between intracellular redox homeostasis and glycogen metabolism [7].

Hepatic ER is involved in different liver functions. The basis of these functions is the hepatic ER membrane-bound enzymatic “tool kit”, frequently combined with transporters, and the different metabolic processes in the luminal compartment of the ER, which require special conditions, that are different from those found in the cytosol [11]. Drug glucuronidation, ascorbate synthesis and glucose production in the liver are glycogenolysis-dependent processes. All of them are catalyzed by membrane-bound ER enzymes with an intraluminal active site (glucose-6-phosphatase, UDP-glucuronosyltransferase isozymes and gulonolactone oxidase) supported by transporters for the membrane permeation of their substrates (glucose-6-phosphate, UDP-glucuronate, gulonolactone). Their products are exported by the hepatocytes. Thus, these export processes are glycogen reserve-dependent [7].

Functional but also subcellular morphological studies have confirmed the connection between the glycogen particle and the ER in a series of hepatic biosynthetic processes [13]. It has been also demonstrated the newly formed glycogen is primarily found in ER-rich regions and remains associated with the latter during glycogen deposition and depletion [14]. There is a shift of glycogen molecules inside the cell depending on the size of the molecule, which depends upon fasting or fed state [15,16,17].

### Alterations in ER Membrane—Glycogen Particle Connection in D-Galactosamine Induced Liver Injury

D-galactosamine is a hepatotoxin that causes the binding between the ER and the glycogen particle to become irreversible. D-galactosamine is transformed through the Leloir pathway of galactose metabolism. This way UDP hexosamines are formed from galactosamine, which are substrates of glycogen synthase. When galactosamine derivatives are incorporated into glycogen the properties of the formed “aminoglycogen” change significantly: it can still connect to the ER, but can no longer detach [18,19,20,21]. The study of liver damage caused by D-galactosamine in rodents gave a very apt example of the possibility of a direct morphological and functional connection between ER membranes and glycogen molecules in liver cells. In the Seventies, liver damage caused by D-galactosamine was called galactosamine hepatitis. At that time, the aim of studies was to develop an experimental hepatitis model without viruses, as administration of this aminosugar led to liver injury, which morphologically resembled viral hepatitis. The hepatic injury induced by D-galactosamine was acute and induced a marked inhibition of hepatic protein synthesis in animal experiments in vivo and also in isolated liver cells in vitro [20]. In contrast to other galactosamine-induced macromolecule synthesis inhibitions, such as inhibition of RNA synthesis, the inhibition of protein synthesis proved to be irreversible [18]. In experiments carried out with biochemical methods “aminoglycogen” was shown to precipitate ribosomes and inhibit amino acid incorporation into proteins in a cell-free system [19]. Special cytoplasmic granules and aggregates appeared in the liver cells of D-galactosamine-treated animals. It turned out that the granules consisted of ribosomes, microsomal membranes and abnormal glycogen, when examined by cyto- and histochemical investigations. Ultrastructural observations demonstrated that the granules were surrounded by membranes of rough endoplasmic reticulum. Electronmicroscopic findings of electron dense clusters of ribosomes and glycogen suggested direct damage to the protein-synthesizing apparatus [21]. In vivo treatment of mice with D-galactosamine caused an alteration of mouse liver microsomal membranes, examined by sucrose gradient centrifugation experiments in the ultracentrifuge [18]. Looking at the results from that time with our current knowledge, it is also clear that the aggregates were degraded by glycophagy via the lysosomal pathway [1]. These experiments suggested, that the relationship between glycogen particles and ER may have a significant role not only in cellular physiology but also in cellular pathology.

## 4. Liver Glycogen Particle–ER Connection in the Maintenance of Blood Glucose Level

As glucose is the only molecule which can be utilized by every cell type in the body, the availability of glucose is of special importance and that is why it is essential for life. Glucose can be formed either via gluconeogenesis, which is independent of glycogen metabolism or via glycogenolysis, which depends on glycogen reserves. In a fed state, the liver secretes glucose originating from glycogenolysis by well-documented procedures. The role of the relationship between hepatic glycogen metabolism and the glucose-6 phosphatase ER enzyme complex in the maintenance of blood glucose level is well-known in detail [7]. The glucose-6-phosphatase enzyme and transporter system is one of the best-known ER membrane protein complexes to which the liver’s ability to secrete glucose is linked [22]. The ability of the liver to supply all the cells of the body with nutrients, with secreted glucose, makes liver glycogen a glucose store for all cells. Pathology and diseases based on enzyme deficiency or dysfunction are also well-known opportunities to learn about normal function. Von Gierke’s disease based on the disturbance of the activity of the glucose-6-phosphatase system causing hypoglycemia is a beautiful and apt example of this as detailed in several textbooks and review papers [22,23].

## 5. Liver Glycogen Particle–ER Connection in Drug Metabolism, Biotransformation

Glycogen particle–ER connection is required for certain drug metabolism processes in the liver. Biotransformation is a very specific part of intermediate metabolism. Most of the substrates of the drug metabolism enzymes, both endobiotics and xenobiotics, are molecules that are in no way connected to the energy transformation of the cell, thus they do not belong to intermediate metabolism by definition. However, biotransformation requires cofactors derived mainly from carbohydrate metabolism, partly from glycogen. Therefore, drug metabolism clearly burdens the turnover of cellular material and energy. Derivatives of glucose are sources of a significant part of the cofactors for drug metabolism; the formation of cofactors frequently interferes with the metabolism of glucose. Glucose production remains the priority. Most of the cofactors are formed at the expense of glucose production from glycogenolysis or gluconeogenesis. This also means that the drug metabolism in the liver and the production of glucose in the liver are opposing processes [24].

Drug metabolism has two phases, catalyzed by different types of enzymes. Reactive intermediates are formed in phase I, the reaction of which are catalyzed mainly by oxygenases. Thereafter these intermediates are conjugated in phase II by several different mechanisms, that are in turn catalyzed by different enzymes. However, the different enzymes catalyzing these different types of reactions are located in the ER membrane or take place in the luminal compartment of the ER [24].

The ER is thus an essential organelle, involved in several processes of biotransformation, and as mentioned before, glycogen particles are the sources of cofactor supply required for conjugation with UDP-glucuronic acid, which is a 2nd phase reaction [25,26]. While the majority of oxygenases and several conjugation enzymes are localized in ER membranes, the orientation of their active sites is different. In the case of UDP glucuronosyltransferases, the active site is intraluminal, while glucuronidations occur in the luminal compartment of the ER [24,25,26].

We have described the dependence of drug glucuronidation on glycogenolysis [12]. Inhibition of glycogenolysis caused by various agents (insulin, fructose, and glucose) decreased glucuronidation in isolated glycogen-containing hepatocytes obtained from fed mice. Glucuronidation was abolished in hepatocytes isolated from glycogen-depleted livers of starved animals. Glycogen particles in the liver are the source of UDP-glucuronic acid for glucuronidation [12,24,27]. Therefore, an increase in biotransformation causes an increased demand for glucuronic acid formed at the expense of glycogen content. That is why increased drug metabolism is a metabolic burden on glycogenolysis in hepatocytes. Different physiological (starvation), and experimental (e.g., galactosamine hepatitis, endotoxin) conditions, which also mimic inflammation or infection can influence liver glucose and drug metabolism simultaneously; glycogenolysis is decreased, glucose secretion is enhanced, and glucuronidation is also decreased, or inhibited [20,24,28]. The preference of hepatic glucose secretion over biotransformation has been shown to occur upon treatment with endoxin and eicosanoid, where glucose secretion was stimulated and drug glucuronidation was depressed at the same time. The local hormone eicosanoids are among the mediators of the hepatocyte-Kupffer cell connection, which is of essential importance for both physiological and pathological liver functions [28].

After glucuronides are synthesized in reactions catalyzed by UDP glucuronyl transferase isozymes in the luminal compartment of the ER, they are secreted into bile from liver cells. Thus, part of the glucose stored in the glycogen particles is exported in form of different glucuronides to the intestinal canal and is excreted from the body through feces. In this way, glucuronidation significantly burdens the glycogen reserves of the liver. This burden depends, on the one hand, on the nature and extent of the intake of xenobiotics of foreign origin (e.g., drugs). On the other hand, endobiotic load, which also burdens the liver’s detoxification system, can also have a significant effect (e.g., certain types of jaundice).

There are metabolic connections between carbohydrate metabolism and biotransformation, where the cofactor supply is also linked to glucose metabolism. but glycogen metabolism is not involved. NADPH is the cofactor of mixed-function oxygenases participating in the oxidation of drugs. NADP can be reduced in the oxidative branch of the pentose phosphate pathway, or at the expense of gluconeogenesis, where NADP reduction is coupled to the oxidation of certain gluconeogenesis intermediates. A relationship between the increase in drug oxidation and the simultaneous depression of hepatic glucose production has been demonstrated under various conditions [24].

In the absence of glucuronidation reactive, unconjugated, hydroxylated intermediates may accumulate. These intermediates significantly influence the redox state of the cell. The 2nd, conjugation phase for detoxification of the reactive intermediates prevents oxidative redox changes caused by the unconjugated intermediates [24,29].

## 6. Glycogen Particles in Antioxidant Defense. Redox Aspects of the ER-Glycogen Particle Connection

Studies on the enzymatic regulation of glycogen metabolism have enriched the world of biochemistry, cell physiology and signal transduction with many great discoveries (cAMP, etc.). The complex and complicated regulation of glycogen metabolism is essential for the regulation of intermediate metabolism. However, as it turns out, the regulation of glycogen metabolism is also related to the regulation of the intracellular redox state in the liver. Besides the well-known hormonal and other regulators of carbohydrate metabolism, important electron carriers, especially water-soluble glutathione and ascorbate, which are essential constituents of antioxidant defense are also involved in the regulation of glycogen metabolism enzymes. Regulation of glycogenolysis in hepatocytes was also shown to be influenced by glutathione. Glutathione (GSH) depletion has been shown to stimulate glycogenolysis in the liver [30]. A decrease in the GSH/GSSG (reduced/oxidized glutathione) ratio causes increased glucuronidation, which is accompanied by stimulated glycogenolysis and elevated UDP-glucose content [31]. The liver plays a central role in the antioxidant defense of the organism, and the role of glutathione in this regulation is fundamental from several points of view [32]. In addition, a characteristic redox environment is required for the optimal functioning of several intraluminal pathways, which are defined by the redox couples of the main electron carriers. The composition, concentration, and redox state of glutathione, pyridine nucleotides, and ascorbic acid are characteristically different from those observed in other subcellular compartments. One of the most characteristic differences is that luminal thiols involving glutathione are present in a more oxidized state compared to the cytosol [33,34,35,36].

**Figure 2:**
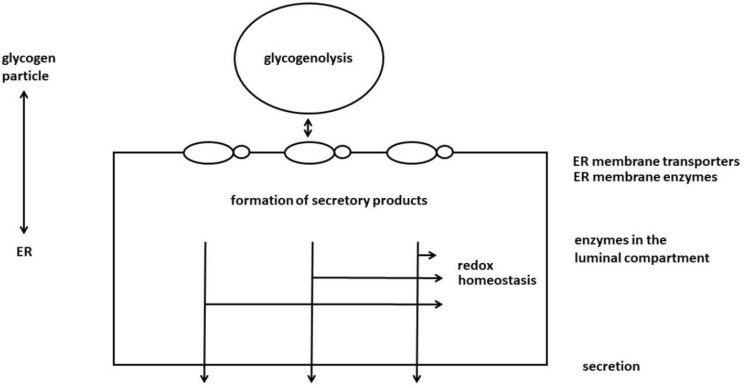
Glycogen particles attach to or detach from the ER membrane. When they connect, then glucose derivatives produced in glycogenolysis enter the luminal compartment of the ER via ER transporters and are further metabolized using the ER membrane enzymes linked to the transporters. In this way, glycogenolysis supplies certain biosynthetic processes in the ER with precursors. Finally, the products of biosynthetic processes are secreted. In addition, the glucose derivatives from glycogenolysis participate in shaping the redox homeostasis of the luminal compartment. Glucose derivatives can enter the pentose phosphate pathway, the operation of which is related to the formation of reduced pyridine nucleotides. The lack of drug glucuronidation affects the amount of GSH in ER. The redox regulation of glycogenolysis connected to the water-soluble antioxidants, GSH and ascorbate can also have various effects.

UDP-glucuronic acid originating from glycogen breakdown is not only the cofactor for glucuronidation [12] but serves at the same time as a precursor for ascorbate synthesis [37]. Ascorbic acid is synthesized from UDP-glucuronic acid in the hexuronic acid pathway in the liver and kidney in animals. It is also well-known that several species, including humans, are unable to form ascorbate as the last enzyme of the hexuronic acid pathway, gulonolactone oxidase has been lost. Still, it is very instructive to keep in mind, that in species which did not lose their ability to synthesize ascorbate, ascorbate production is dependent on glycogen stores [37,38]. The source of the cell’s own ascorbate production is glycogenolysis. Increase in glycogenolysis stimulates ascorbate formation, while a decrease inhibits it in mice [38]. It is also noteworthy that gulonolactone oxidase, the key enzyme in ascorbate synthesis is also a microsomal enzyme [39], and ascorbate can be also secreted from the liver similarly to glucose, glucuronides, whose secretions from hepatocytes were connected to glycogenolysis [7] (Figure 2).

This way glycogen metabolism might be involved in the complicated relationship between the two water-soluble antioxidants in the antioxidant defense, namely in several cases they can substitute each other, and dehydroascorbate can be reduced at the expense of reduced glutathione [40].

Both ascorbate and glutathione participate not only in the maintenance of the oxidative redox state of the luminal compartment, but also in several very important redox processes, such as protein folding, etc. [36]. It is also noted that conjugation with glutathione is a fundamental phase II reaction of biotransformation.

## 7. Actual Metabolic State of the Liver Is Dependent on Hepatic Glycogen Content. Depletion of Liver Glycogen in Starvation

Glycogen content is considered to be a sensitive marker of the actual metabolic state of the liver. The transformation of several liver functions during starvation, which is a physiological condition that accurately shows the role of glycogen reserves both in liver function and also in the regulation of these functions. In an experimental fasting-refeeding cycle-intensive glycogenolysis is associated with the proliferation of the smooth ER [41].

The early postnatal period is a good physiological example to show the function of glycogen stores and the significance of the simultaneous intermediate metabolism and ER membrane-bound enzyme inductions [42,43]. Glycogenolysis and the glycogenolysis-dependent ER pathways seem to be activated together with the proliferation of the smooth ER [44]., the induction of enzymes such as the glucose-6-phosphatase system, several UDP-glucuronosyltransferase isoenzymes, and gulonolactone oxidase [43].

In a similar fashion, starvation also has effects on other connections between drug- and carbohydrate metabolism. In well fed state pentose phosphate pathway is sufficient for NADPH production required for cofactor supply for phase 1 oxidation biotransformation reactions, However, in starvation, in case of increased cofactor demand raised by the necessity of increased drug exposition, NADPH is produced at the expense of gluconeogenesis, as intermediates of gluconeogenesis (malate, glucose 6-phosphate), are shifted away from the gluconeogenic pathway toward NADPH generating reactions [24].

Glucuronidation is important for the termination of the action of several drugs, and prevents the accumulation of potentially dangerous toxic intermediates of biotransformation. It is well known that since starvation reduces glucuronidation, bilirubin, the most important endobiotic substrate of biotransformation cannot conjugate either. This is known as fasting hyperbilirubinemia [45,46].

As drugs are eliminated through different phase I, and phase II reactions of drug metabolism in the liver, if the very reactive intermediates formed in phase I are not conjugated, they can enter into redox cycles and can be involved in the free radical formation, which causes redox stress [47]. Furthermore, they can attack macromolecules, such as DNA or proteins and can cause hepatocellular toxicity, ER stress, mutations and also chemical carcinogenesis, etc. [28,47]. If liver glycogen stores are depleted due to starvation and UDP-glucuronic acid cannot be formed, glucuronidation is decreased. In the lack of glucuronidation other phase II reactions may replace glucuronate conjugation (e.g., conjugation with glutathione), but the reactive products of the oxidative phase I, unconjugated hydroxylated intermediates may also accumulate [24].

## 8. Pathological Aspects of Deprivation of Liver Glycogen—DILI in Starvation

Paracetamol overdose toxicity is a good example of altered drug metabolism due to the lack of UDP-glucuronic acid, as a glucuronidation cofactor in starvation. Paracetamol (acetaminophen, N-acetyl-*p*-aminophenol, APAP) is the most common painkiller with an antifebrile effect, but at the same time it is a dose-dependent hepatotoxin. Reactive derivatives formed in the oxidation of APAP are conjugated with UDP-glucuronic acid [48]. Paracetamol overdose causes drug-induced liver injury (DILI); APAP is responsible for approximately 50% of all acute liver failure cases in the US and UK [49]. It is noteworthy, that in the DILIN (North American Drug Induced Liver Injury Network), the presentation of DILI was accompanied in 70% of cases by jaundice [49]. Oxidative mitochondrial damage and ER stress are underlying APAP toxicity at the hepatocellular level, which are significantly aggravated in starvation when glycogen stores are depleted, and reactive derivatives of APAP cannot be conjugated [48,50,51,52].

## 9. Conclusions

ER is a separate intracellular metabolic compartment, which has an extracellular-like environment and is part of the secretory pathway in the liver [9,34,35,36]. Transport of the required intermediates, cofactors and precursors across the ER membrane is essential for several ER-specific metabolic processes, and glycogenolysis is one of the sources of such molecules. Therefore, the glycogen particle–ER connection is decisive in maintaining the metabolic homeostasis of the liver cell. The depletion of the glycogen store and the lack of the glucogenic reserve transform the metabolism of the liver cell. The continuous change of liver glycogen content depends partly on changes in external conditions—Starvation versus food intake, xenobiotic exposure, etc. [10,11,24]. Therefore, the liver glycogen particle, itself is an important tool for adaptation to environmental changes. Redox conditions in the luminal compartment of the ER are also essential for several processes, such as oxidative protein folding, etc. [10,11,36,47].

Further research is needed to understand certain elements of the relationship between the regulation of glycogen degradation and the hepatocellular intraluminal redox state. In addition, glycogen metabolism has recently become one of the targets of antitumor therapy [53]. One explanation for this is the relationship between glycogen metabolism and redox homeostasis.
